# Smartphone Apps for Food Purchase Choices: Scoping Review of Designs, Opportunities, and Challenges

**DOI:** 10.2196/45904

**Published:** 2024-03-06

**Authors:** Remco Benthem de Grave, Christopher N Bull, Diogo Monjardino de Souza Monteiro, Eleni Margariti, Gareth McMurchy, Joseph William Hutchinson, Jan David Smeddinck

**Affiliations:** 1 Open Lab School of Computing Newcastle University Newcastle upon Tyne United Kingdom; 2 Centre for Rural Economy School of Natural and Environmental Sciences Newcastle University Newcastle upon Tyne United Kingdom; 3 Ludwig Boltzmann Institute for Digital Health and Prevention Salzburg Austria; 4 Ludwig Maximilian University Munich Germany

**Keywords:** behavior change, mobile apps, food choices, grocery shopping, sustainability, healthy eating, digital health, mobile phone

## Abstract

**Background:**

Smartphone apps can aid consumers in making healthier and more sustainable food purchases. However, there is still a limited understanding of the different app design approaches and their impact on food purchase choices. An overview of existing food purchase choice apps and an understanding of common challenges can help speed up effective future developments.

**Objective:**

We examined the academic literature on food purchase choice apps and provided an overview of the design characteristics, opportunities, and challenges for effective implementation. Thus, we contribute to an understanding of how technologies can effectively improve food purchase choice behavior and provide recommendations for future design efforts.

**Methods:**

Following the PRISMA-ScR (Preferred Reporting Items for Systematic Reviews and Meta-Analyses extension for Scoping Reviews) guidelines, we considered peer-reviewed literature on food purchase choice apps within IEEE Xplore, PubMed, Scopus, and ScienceDirect. We inductively coded and summarized design characteristics. Opportunities and challenges were addressed from both quantitative and qualitative perspectives. From the quantitative perspective, we coded and summarized outcomes of comparative evaluation trials. From the qualitative perspective, we performed a qualitative content analysis of commonly discussed opportunities and challenges.

**Results:**

We retrieved 55 articles, identified 46 unique apps, and grouped them into 5 distinct app types. Each app type supports a specific purchase choice stage and shares a common functional design. Most apps support the product selection stage (selection apps; 27/46, 59%), commonly by scanning the barcode and displaying a nutritional rating. In total, 73% (8/11) of the evaluation trials reported significant findings and indicated the potential of food purchase choice apps to support behavior change. However, relatively few evaluations covered the selection app type, and these studies showed mixed results. We found a common opportunity in apps contributing to learning (knowledge gain), whereas infrequent engagement presents a common challenge. The latter was associated with perceived burden of use, trust, and performance as well as with learning. In addition, there were technical challenges in establishing comprehensive product information databases or achieving performance accuracy with advanced identification methods such as image recognition.

**Conclusions:**

Our findings suggest that designs of food purchase choice apps do not encourage repeated use or long-term adoption, compromising the effectiveness of behavior change through nudging. However, we found that smartphone apps can enhance learning, which plays an important role in behavior change. Compared with nudging as a mechanism for behavior change, this mechanism is less dependent on continued use. We argue that designs that optimize for learning within each interaction have a better chance of achieving behavior change. This review concludes with design recommendations, suggesting that food purchase choice app designers anticipate the possibility of early abandonment as part of their design process and design apps that optimize the learning experience.

## Introduction

### Background

Recent reports suggest that current consumer patterns, particularly in Western societies, have a significant impact on health and pose a considerable burden on the natural environment [1,2]. There is increasing evidence that the food production systems and processes enabling current diets contribute to biodiversity losses, water scarcity, and climate change [2,3]. In addition, obesity and food-related diseases affect a significant proportion of the global population [4], leading to an increasing economic and social burden at both the individual and aggregate levels [1,5,6].

Although many people claim to have values that align with healthy and environmentally sustainable diets [7], actual consumption patterns are often inconsistent with such stated preferences [8,9]. Research has found that people often struggle to select suitable products to support a healthy or sustainable diet [10]. Diet choices are affected by factors such as broader values, preferences, prices, availability, social pressure, and socioeconomic constraints [10,11]. This frequently leads to inconsistencies and compromises between competing priorities [12]. Moreover, bounded rationality (ie, the limited capacity for rational decision-making) and heuristic biases (ie, rational errors in the way we make quick decisions) often lead to suboptimal choices.

Digital technology—particularly smartphone technology—can support people in making suitable food purchases [13,14] that align with their values. Smartphone technology (hereafter referred to as *apps* but not excluding any smartphone technology that is not strictly considered an app) can provide rapid access to information, summarize large amounts of data, and present these in personally meaningful ways. With smartphones being omnipresent, these capabilities are available at nearly any time and place, alleviating the need for retailers to make financial investments that could hold back the implementation of alternative systems [15,16]. In the last decade, several software and hardware mobile technologies have emerged to support consumers in making better (ie, healthier and sustainable) diet choices. This is particularly the case for healthy diet support apps, which have received much interest in recent years, particularly in the form of diet-tracking technologies [13,17-19].

Although a large body of research has examined digital technology for consumption tracking of different products [17-20], research on smartphone apps that support food purchase choices is still limited [13,14]. Responding to Flaherty et al [13] and Chan et al [14], who call for further research on the development of food purchase choice apps and to support the development of effective food purchase choice app designs, we conducted a comprehensive review of the research on such developments to date.

### Related Reviews

Our scoping review complements previous reviews. Mauch et al [21] reviewed popular apps for healthy food provision available on the Android and iOS app stores. They downloaded 51 apps, assessed the app quality (following the Mobile App Rating Scale [22]), and identified the behavior change techniques (BCTs) present in the design. They classified most apps as recipe, meal-planning, or shopping list apps. In total, 2 were classified as food choice apps. They found that the apps covered relatively few BCTs and that, although the apps generally scored well on functionality, they scored poorly on engagement. They recommended that future developments use a range of features to simplify healthy food shopping and maximize the use of BCTs.

Chan et al [14] conducted a systematic review to assess the efficacy of point-of-sale nutritional information interventions. They included 26 papers that reported comparative evaluations, 5 of which used digital technology as an intervention medium. In total, 3 of the 5 digital technology intervention studies showed a positive health impact, and the authors concluded that digital point-of-sale information interventions could improve healthy food purchasing. However, they noted that, compared with shelf labels, the requirement to scan products to retrieve information may have posed a barrier that led to inconsistency in the results. They found no relationship between intervention effectiveness and the number of identified BCTs.

### Objectives

To the best of our knowledge, the reviews by Mauch et al [21] and Chan et al [14] are the only ones of food purchase choice apps to date. However, they are incomplete. Mauch et al [21] provided a catalog of publicly available health and diet apps without much appraisal of their use. Chan et al [14] surveyed evaluations of food purchase choice apps but limited the scope of their review to 5 studies that followed a comparative evaluation design. Thus, our review aimed to complement their work by compiling a comprehensive overview of the academic literature on apps for food purchase choice published to date. Specifically, we aimed to (1) describe common design characteristics of the retrieved apps and (2) describe opportunities and challenges to effective implementation as they are observed in the literature.

To address these objectives, we chose to conduct a scoping review. Similar to meta-analyses, scoping reviews are systematic, yet they are less restrictive in scope. They are suitable for mapping the knowledge on a topic and the characteristics of the evidence [23], which makes them well suited for our aims.

This work provides an overview of existing food purchase choice app characteristics, unpacking how different design approaches might affect food purchase choices. Moreover, it frames common challenges in the implementation of these apps. Finally, it frames design considerations that could support future effective food purchase choice app developments**.**

## Methods

### Overview

We conducted a scoping review of published literature on smartphone-compatible digital technologies for food purchase choices (food purchase choice apps). The review followed the guidelines covered by the PRISMA-ScR (Preferred Reporting Items for Systematic Reviews and Meta-Analyses extension for Scoping Reviews; Multimedia Appendix 1 [23]).

### Information Sources

We searched for peer-reviewed articles in 4 libraries that were selected to provide coverage of digital health and well-being technologies from an empirical as well as technical perspective: IEEE Xplore, PubMed, Scopus—which covers the ACM Digital Library—and ScienceDirect. The most recent database search was conducted on July 10, 2023. Together, these libraries cover a broad range of literature, with research on mature as well as emerging technologies and publications from the fields of behavioral science, economics, and computer science. We further scanned the included studies for references to articles that would meet our inclusion criteria.

### Search

We searched the 4 libraries using the following keyword combination: “(mobile OR smartphone OR app) AND (purchase OR shopping) AND (food OR grocery OR supermarket).” We limited our search to articles published from 2008 onward. We chose this date limit in consideration of the introduction of the iPhone—the first widely adopted consumer smartphone with a noteworthy ecosystem of apps—in January 2007 and the time required for app development to pick up.

### Selection

For selecting the articles, we applied a stepwise process that involved 3 reviewers (RBG, JH, and GM). RBG exported the search results from the individual database searches, imported them into the article selection tool Rayyan (Rayyan Systems Inc) [24], and removed duplicates. We then proceeded with the selection of articles according to the eligibility criteria as described in Textbox 1. First, we filtered articles based on title and—if the title left uncertainty about inclusion or exclusion—abstract. RBG scanned the full set of articles. JH and GM scanned approximately half of the set each. This way, each article was scanned independently by at least 2 authors. We then compared the results and discussed any differences until we reached a mutual agreement. RBG then read the full texts of all the articles that remained for another round of filtering. JH and GM together read 17 full-text articles so that a subset of full-text articles was read and selected by multiple researchers. Any differences in selection were discussed until a mutual agreement was reached.

**Eligibility criteria for article selection.**

**Inclusion criteria** **(all must apply)**

- App design characteristics:
  Designed for or compatible with smartphones (such as smartphone apps, web applications, or SMS text messaging)
  Designed to support grocery choices by considering noncommercial product information types such as nutritional value and social or environmental impact
- Article characteristics:
  Primary works in peer-reviewed journals

**Exclusion criteria** **(any of these resulted in exclusion)**

- App design characteristics:
  Does not address diet choices
  Does not have an apparent influence on the decision process (eg, if the technology focuses on automatic billing)
  Is exclusively designed for web-based grocery purchases (most purchases are still made in brick-and-mortar grocery stores [25], and interventions for brick-and-mortar and web stores involve rather different interaction challenges; designs for purchases in brick-and-mortar stores need to address transitions between the physical and digital spaces [26,27], whereas designs for web-based purchases do not need to address this transition)
  Recipe recommenders (that recommend recipes rather than products)
  Diet-tracking apps (unless specifically tracking purchases rather than consumption)
  Designed specifically to provide support regarding physical disabilities (eg, mobility and eyesight disabilities)
  Designed around exclusive programs (eg, exclusive voucher programs available only in restricted areas)
  Technology purely designed and intended as a study support tool
- Article characteristics:
  Literature reviews (in case relevant reviews were retrieved through the literature search, they were covered as related work in the Introduction section of this paper)
  Not in English

### Data Charting

The data charting process (also referred to as data extraction) was iterative, and the coding of several items was inductive.

#### Step 1: Open Coding

Open coding was performed by following an instruction manual and the codes were inserted into a Microsoft Excel (Microsoft Corp) spreadsheet that served as the charting form. The instruction manual was drafted by RBG and subsequently verified for comprehensiveness and clarity of instruction through duplicate coding of a test set of 7 articles by RBG, JH, and GM. For verification, the 3 coders met to compare and discuss differences in coding, and minor adjustments to the instruction manual were made. The 3 coders then continued to code the remaining articles. JH and GM each coded another 5 articles each, and RBG coded the remaining articles. Open coding at this stage aimed to cover relevant information at a high level of detail such that this could facilitate the inductive process. Variables that were coded at this stage were app variables (description of the app, including the user interaction, functions, required technical infrastructure, and considered product information), evaluation variables (study design, participants, findings, and reported challenges and opportunities), and article variables (publisher and publication year). In case the same app was discussed in multiple articles, the information for the app variables was combined.

#### Step 2: Collaborative Identification of Patterns

After completion of open coding, the coders held a workshop meeting to identify patterns in several of the data items from step 1 (description of the app, functions, findings, and reported challenges and opportunities) to determine app types, a harmonized collection of functions, and topics of opportunities and challenges. Miro (RealtimeBoard, Inc) [28], a digital whiteboard for collaborative note taking, was used for this workshop. Post-It notes with the initial codes for several of the data items (excluding the article items) were added to the whiteboard. A complete set of these notes was copied for each of the 3 coders, which they could reorganize to aid in the process of exploring and identifying patterns. Workshop participants also had access to a Microsoft PowerPoint (Microsoft Corp) slide deck with images and descriptions of each app and the spreadsheet with the complete coding from step 1. Pattern exploration was performed in 6 rounds. In each round, a subset of step 1 items was explored, first independently by each of the participants and then collectively.

#### Step 3: Final Coding

RBG updated the charting form with the outcomes of the workshop. Several data items were replaced such that the new data items aligned with the patterns from the workshop. In addition, the full texts of the articles were scanned for references to the patterns of opportunities and challenges that were identified in the workshop (also known as *indirect* opportunities and challenges).

### Data Items

The final set of charted data items is outlined in the following sections.

#### Article Characteristics

We charted publication year and publisher. Classification of study types was coded according to the classification adapted from the study by Bardus et al [29]. We classified article types as (1) *design and development* (articles that described the design of an app), (2) *feasibility* (articles reporting procedural outcomes such as use, acceptance, and retention, as well as articles reporting outcomes of experiments that were performed under restrictive conditions [eg, laboratory setting or a predetermined selection of products to choose from]), (3) *evaluation* (reporting the effectiveness of a technology intervention trial on food purchase choice), and (4) process evaluations and causal-comparative studies (*process evaluations* in short; reporting effects on sociocognitive factors that are related to food purchase choice or reporting on comparisons with alternative interventions). A fifth type was reserved for *app store review*s. Combination types were also possible (eg, an article could cover both a feasibility and an evaluation study and would then be assigned both codes).

#### Design Characteristics

We recorded the *app name*. The variables *app type* and *functions* were charted in correspondence with the patterns that evolved from the workshop (see the *Data Charting* section, step 2). *Product information* represents the type of information that the app uses to support product choice. This covers nutritional information (eg, calories, allergens, and macro- and micronutrients), diet balance (or food group; information on food groups of purchases concerning recommendations for a balanced diet, such as MyPlate [30] or the Eatwell Guide [31]), environmental impact (eg, carbon footprint and food miles), and societal impact. *Retailer dependency* was classified as “dependent” in case the article mentioned the use of data infrastructure that is controlled by the retailer (eg, the retailer product database, loyalty card data, or beacons for indoor navigation), “independent” in case the article stated that only crowdsourced or open-access data were used, or “unknown” when this information was unavailable.

#### Study Characteristics (for Evaluation Studies Only)

We recorded the *study design* (ie, cohort or control group design), evaluation period (*duration*), number of participants in each study group (*participants*), and primary findings (*findings*). The topics of *opportunities and challenges* were coded in correspondence to the patterns from the workshop. Here, we distinguished between *direct* (reported as a challenge or opportunity in the article) and *indirect* (not reported as a challenge to the design and identified in the second pass of the article).

### Synthesis of Results

We counted the frequencies at which the values were observed and provided narrative summaries of the findings. Specifically, to synthesize the findings on apps, we grouped observations (ie, apps) by app type and summarized the design characteristics for each group. We supplemented this with a narrative summary in which we focused on common patterns and salient exceptions to those patterns. Furthermore, we visualized the frequency of functions per app type using a bar chart.

Next, we investigated the opportunities and challenges from a quantitative and qualitative perspective. From the quantitative perspective, we counted significant and insignificant primary evaluation outcomes and provided a narrative summary of dominant patterns in the results. From the qualitative perspective, we provided a content analysis of the charted opportunities and challenges, meaning that we tabulated an overview of counted topics of opportunities and challenges and elaborated on these topics using a narrative summary that was supported by specific observations in the articles.

## Results

### Overview of the Included Articles

**Figure 1:**
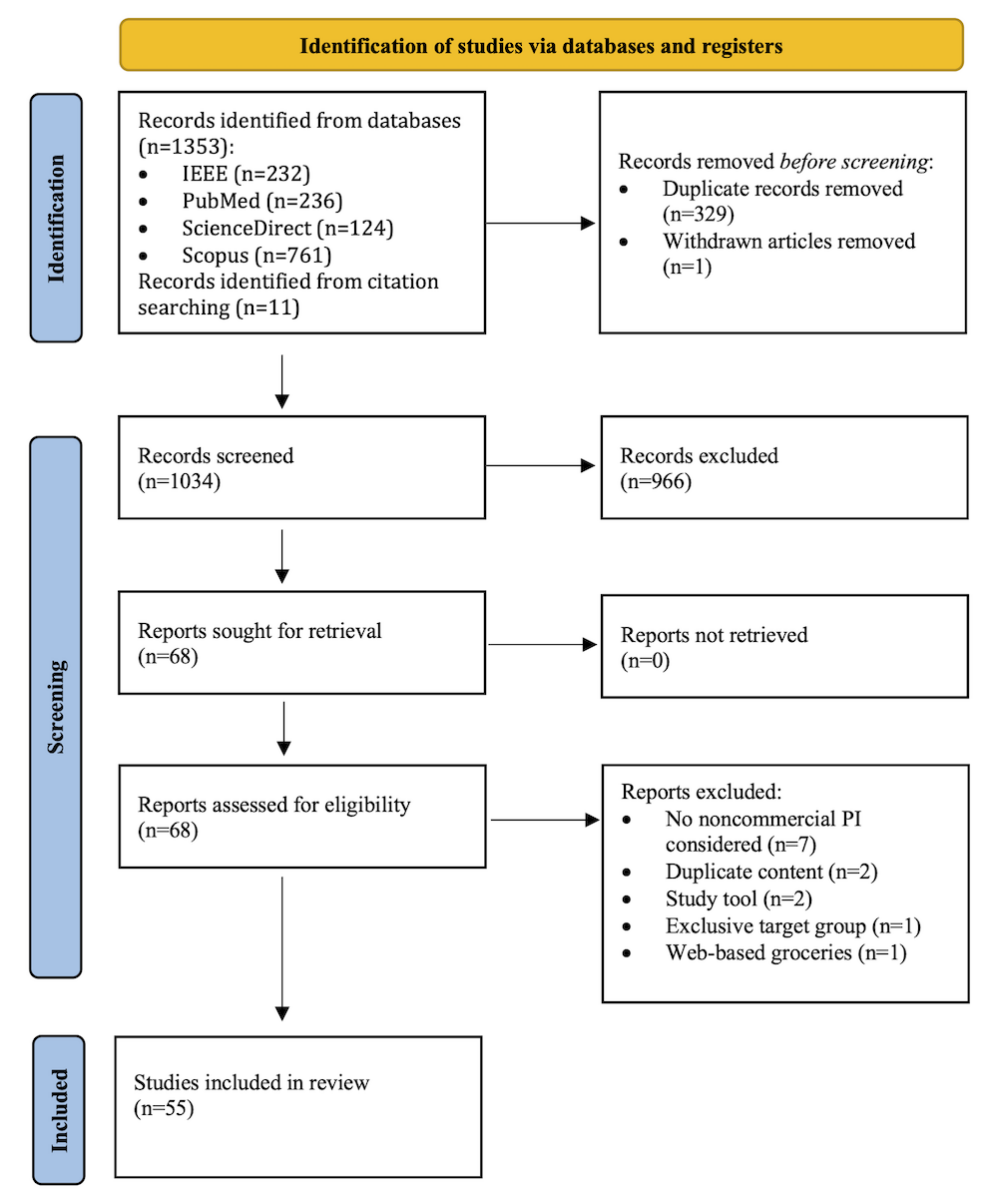
Results of article search and screening. PI: product information.

The database searches returned a total of 1353 articles. Another 11 articles were included from references. After screening, we retained 4.03% (55/1364) of the articles for this review. Figure 1 provides an overview of the number of articles excluded at each step of the selection process and the reasons.

Among the 55 articles that remained after filtering, we identified 41 (75%) design and development studies, 32 (58%) feasibility studies, 13 (24%) evaluations, and 3 (5%) process evaluations. In total, 4% (2/55) of the articles were classified as app store reviews. These counts do not add up to 55 as articles often covered a combination of classifications. The dominant publication venues were *IEEE* (engineering; 13/55, 24%), *ACM* (computer science; 13/55, 24%), *Elsevier* (7/55, 13%), *JMIR* (5/55, 9%), and *Springer* (5/55, 9%). A complete overview of the included publications is available in Multimedia Appendix 2 [9,12,13,16,21,32-81].

### Summary of Apps

#### Overview

We charted the characteristics of 46 unique food purchase choice apps (see Multimedia Appendix 3 [9,12,16,33-81] for a complete overview). A total of 5% (3/55) of the articles [13,21,32] provided insufficient information on their covered apps for us to chart them. We identified 9 main functions. For 3 of these, we distinguished different implementations of the functions, creating a total of 21 different function labels (see Multimedia Appendix 4 [9,27,33-36,40,41,44,48,49,52,71,82] for descriptions). Most apps provided health-relevant information (37/46, 80%), with 70% (32/46) of the apps returning nutritional content and 13% (6/46) of the apps providing information on diet balance (1/46, 2% provided information on both). A total of 26% (12/46) of the apps provided information on environmental impact. Other product qualities included societal impact (3/46, 7%), product freshness (1/46, 2%), and product authenticity (whether the provided origin and product type were genuine; 1/46, 2%). Approximately one-quarter (12/46, 26%) of the apps were found to be dependent on the retailer.

**Figure 2:**
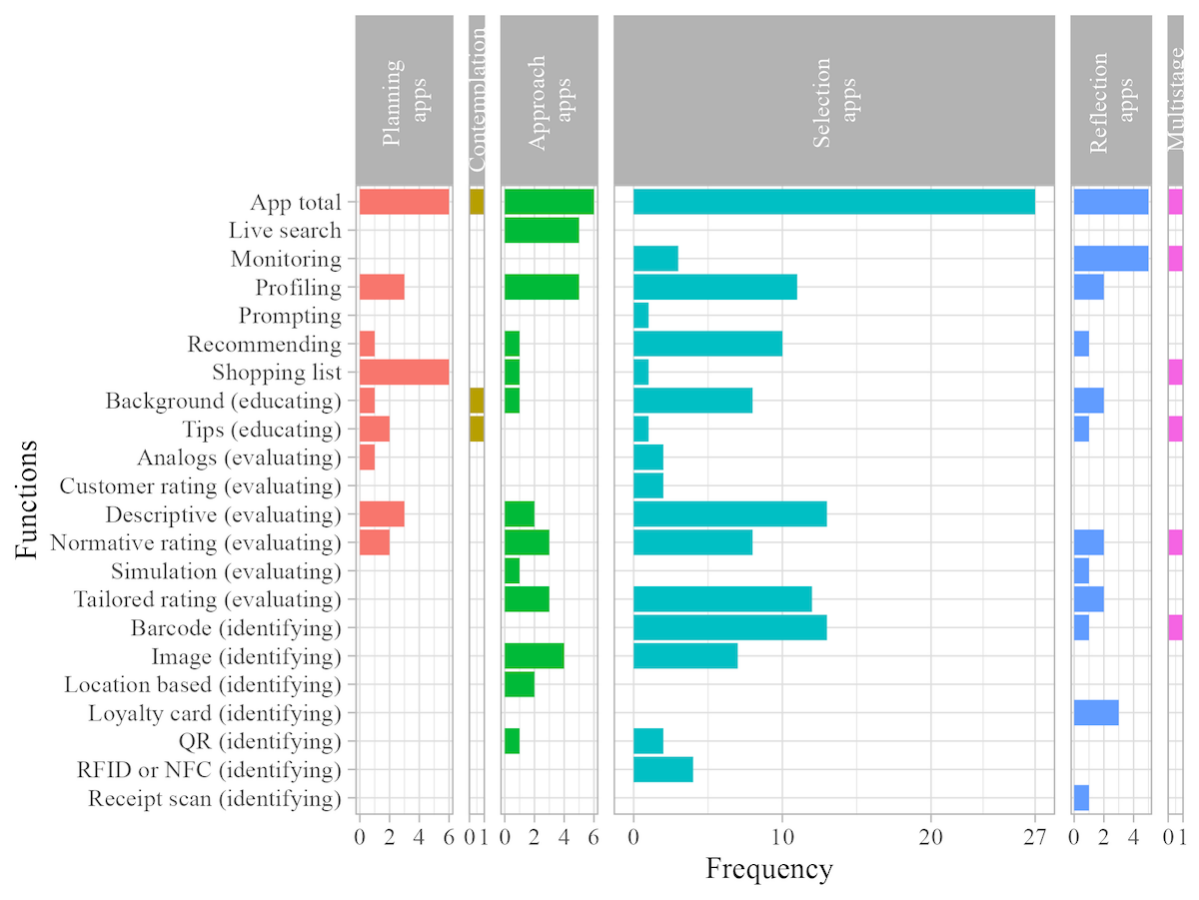
Frequency of functions by app type. Separate panels for each app type are scaled to the total number of apps within the app type. NFC: near-field communication; RFID: radiofrequency identification.

We categorized the apps into 6 app types. We observed that all—except one [33]—of the covered apps (45/46, 98%) provided support for a distinct moment or stage regarding product choice. Therefore, we named each app type according to the stage that it addressed. We found apps that addressed (in chronological order) structured purchase *planning* (7/46, 15%), *contemplation* (2/46, 4%), *approaching the product* (6/46, 13%), physically *selecting the product* (27/46, 59%), and *reflecting* on the purchases (7/46, 15%). A total of 2% (1/46) of the apps addressed a combination of these stages (the planning, contemplation, selection, and reflection types. For calculations and graphical mappings, we did not count this toward the individual app types. In those cases, we included a separate multistage app type). In the following sections, we describe these app types along with common functions (Figure 2; an overview of function descriptions is provided in Multimedia Appendix 4) and examples.

#### Purchase Planning Apps

These apps support the planning for the use of a shopping list. This can be done by automatically generating a shopping list [34], suggesting a goal for the shopping list content [33,35], or providing feedback about product characteristics within the shopping list [36,37].

For example, Hedin et al [36] used an autocomplete algorithm that lists food items from a carbon footprint database while the user types. The carbon footprint value per 100 g is then presented next to the list item.

#### Contemplation Apps

The contemplation apps have a mostly practical educational function and provide knowledge and tips that help the user make decisions about food purchases while shopping.

For example, Bangia et al [38] introduced a podcast series that informs the listener about the benefits of omega-3, the types of products in which it is found, and practical considerations such as the affordability of different forms of omega-3. The podcast series has short episodes, one each covering products that are typically found in the same shopping aisle so that it can be played when visiting the specific aisle.

#### Approach Apps

Product approach apps use the live search function to provide information about nearby products. Options are visualized on the device screen together with suitability ratings for each product. These apps were found to provide nutritional and environmental but not diet balance information.

For example, the app described by Isley et al [39] uses augmented reality to project semitransparent traffic-light color frames and A-to-F tailored ratings over breakfast cereal boxes as the user views the respective shelf section through the phone camera.

Special cases were 4% (2/46) of the apps, which used indoor position data to provide the user with information about nearby products but relied on the retailer for these data.

#### Selection Apps

Selection apps provide the user with an evaluation of the product after it has been identified. *Barcode scanning* (13/46, 28%) is the most common type of identification. *Normative ratings* (8/46, 17%) and *tailored ratings* (12/46, 26%) are the most common types of evaluations. Many of these apps also recommend alternative products from the same product category (10/46, 22%; eg, the category of milk and dairy-free milk alternatives).

For example, upon barcode scanning, App 30 (FoodSwitch) [40] shows the product name and traffic-light nutrition label for the identified product and lists same-category alternatives ordered from more to less healthy below the identified product.

The following were special cases. Although selection apps rely on the active pursuit of product information, one of the identified selection apps makes an exception and allows the user to be passive in the information pursuit. Customers use the retailer-dependent App 22 (Dirk app) [41] to scan articles as they go around the store as part of a checkout-free automated payment process. In case the user scans an unhealthy item, the app provides a pop-up with information and an alternative.

In addition, one selection app, App 41 (Nutriflect Mobile) [42], is designed to be used in combination with a reflection app, App 40 (Nutriflect Home), that records the diet balance in food purchases. This is the only selection app that provides feedback on diet balance (see also the visual in Multimedia Appendix 5).

#### Reflection Apps

These apps rely on monitoring purchases to provide the user with feedback on an aggregated data visualization. Some of these apps are intended to be used in the store [16,33,35], tracking items as they are added to the shopping basket and providing the user with the opportunity to make adjustments before leaving the store. Other apps provide feedback based on a record of purchases (eg, from loyalty card data) and support intention formation for the next purchase.

For example, Bird et al [16] let the user scan the barcodes of products as they were added to the shopping basket to receive diet balance information. The app interface provided visual feedback on the content of the basket according to 5 food groups (grains, meat, dairy, fruits and vegetables, and treats) through a pie chart. The size of each slice of the pie chart was proportional to the recommended number of purchases for that food group, and each slice could be partly filled using, for example, light green to represent the remaining amount to be added to the basket and dark green to represent the amount already added.

#### Multistage Apps

One app was found to address multiple stages of the purchase process. Bomfim and Wallace [33] and Bomfim et al [35] proposed an app, App 44 (PBGA), that covers the planning, contemplation, selection, and reflection stages to support users in achieving a balanced diet. App 44 (PBGA) supports users in creating a shopping list in line with a healthy diet balance (planning), provides users with tips for identifying healthy options in various aisles (contemplation), provides nutrient information upon scanning the barcode of a product (selection), and monitors the scanned items to recommend purchases in each of 5 food groups.

### Challenges and Opportunities

In the following sections, we explore the opportunities and challenges associated with the effective implementation of food purchase choice apps. For this purpose, we summarize the results of the evaluation studies and report a qualitative content analysis of common challenges.

#### Study Outcomes

Table 1 provides an overview of the 11 evaluation studies. These evaluation studies cover 22% (10/46) of the apps: 7% (2/27) of the selection apps (lowest) and all the reflection apps (7/7, 100%; highest). In total, 72% (8/11) of the studies reported a significant behavioral impact. However, mixed results were observed for the 2 evaluated selection apps. Otherwise, no clear patterns between app type and study outcome could be observed. A total of 27% (3/11) of the studies reported outcomes (findings) toward a diet balance, all showing significant results (see also Multimedia Appendix 6 [16,38,41-49] for grouping of evaluations by product information).

Table 2 provides an overview of the 6 process evaluations and causal-comparative studies. Knowledge gain was the most commonly reported sociocognitive outcome factor (4/6, 67%). All these studies reported significant knowledge gain.

**Table 1:** Summary of evaluation studies organized by app type.

App type and name			Design		Duration	Participants		Findings
**Planning apps**								
	App 39 (MyNutriCart) [43]	Cohort		8 wk		27 participants	Significant healthier diet balance of purchases	
**Contemplation apps**								
	App 5 [38]	Cohort		6 mo		251 participants	Significant increase in omega-3–rich purchases	
**Approach apps**								
	App 8 [44]	Cohort		7 mo		69 participants in the intervention group and 323 participants in the control group	Significant increase in higher-rated purchases	
**Selection apps**								
	App 22 (Dirk app) [41]	3 UI^a^ variations (1 × presenting the alternative without information about its healthiness and 2 × with variations of information that indicate that the presented item concerns a healthier alternative) vs control		5 wk		1783 scans^b^	Significantly healthier purchases when presenting alternatives without health information and nonsignificantly healthier when adding health information	
	App 30 (FoodSwitch) [45]	App vs no app		4 wk		33 participants in the intervention group and 33 participants in the control group	Significant reduction in sodium concentration in purchases	
	App 30 (FoodSwitch) [46]	App+reduced-sodium salt vs no app		12 wk		84 participants in the intervention group and 84 participants in the control group	Nonsignificant change in sodium intake	
**Reflection apps**								
	App 13 [47]	Cohort		2 wk		31 participants	Nonsignificant change in the healthiness of purchases	
	App 25 (EcoPanel) [48]	App vs no app		5 mo		65 participants in the intervention group and 2587 participants in the control group	Significant increase in organic purchases	
	App 32 (FutureMe) [49]	App+future-self simulation vs app without		12 wk		42 participants in the intervention group and 53 participants in the control group	Nonsignificant difference in the healthiness of purchases	
	App 35 (Healthy Shopping App) [16]	Cohort		4 wk		7 participants	Significantly healthier diet balance of purchases	
	App 40 (Nutriflect Home) [42]	Cohort		4 wk		21 participants	Significantly healthier diet balance of purchases	

^a^UI: user interface.

^b^Participant counts are unknown. Only the number of scans that triggered information prompts was available.

**Table 2:** Summary of process evaluations and causal-comparative studies.

App type and name		Design	Duration	Participants	Findings
**Planning apps**					
	App 33 (GreenCobra) [36]	Cohort	2 wk	30 participants	Significant knowledge gain
	App 37 (MFG^a^) [35]	Cohort	3 wk	12 participants int eh control group	Significant knowledge gain
	App 39 (MyNutriCart) [43]	App vs education	8 wk	27 participants in the intervention group and 24 participants in the control group	Nonsignificant difference in healthiness of purchases vs education
**Selection apps**					
	App 26 (EDO app) [50]	Cohort	12 wk	143 participants	Significant knowledge gain and significant gain in self-efficacy
**Multistage apps**					
	App 44 (PBGA^b^) [35]	Cohort^c^	3 wk	12 participants in the intervention group and 12 participants in the control group	Significant knowledge gain
	App 44 (PBGA) [35]	App 44 (PBGA) vs App 37 (MFG)	3 wk	12 participants in the intervention group and 12 participants in the control group	Significant reduction in ultraprocessed food purchases

^a^MFG: My Food Guide.

^b^PBGA: Pirate Bridgitte’s Grocery Adventure.

^c^Knowledge gain was assessed using a pre- and posttest design, whereas behavior change was assessed by comparing the effects of different apps. Therefore, we list the results in separate table rows even though the 2 evaluations were performed in the same study.

#### Qualitative Content Analysis of Challenges and Opportunities

##### Overview

Table 3 provides the counts of common topics regarding challenges and opportunities as they were identified through a qualitative content analysis (Multimedia Appendix 7 [12,13,16,21,32,35-42,44-60,62-67,69,76,79,81]). We elaborate on these topics in the following sections.

**Table 3:** Common challenge topics found in the studies.

Topic		Direct^a^	Indirect^b^	Total
**Behavior change**		8	10	18
	Barriers to change	5	4	9
**Interaction**		23	20	43
	Engagement	12	2	14
	Trust	5	7	12
	Burden of use	5	4	9
	Learning	1	7	8
**Technical feasibility**		13	7	20
	Source data^c^	8	3	11
	Performance	5	4	9

^a^The authors of the publication recorded the topic as a challenge to the implementation of the app.

^b^The authors addressed the topic but not directly as a challenge to the implementation of the app.

^c^The data that underlie the information that is viewed on the app. This is typically a database of product information.

#### Behavior Change Challenges

##### Barriers to Change

Behavior change is hard, and participants struggle to achieve diet goals [16,35]. Conflicting priorities complicate adoption of changes [12,36,51]. The user may not like the suggested products [51] or perceive the pursuit of adopting changes as too time-consuming [12,36]. Participants look for ways to prioritize their efforts but may find support for this lacking [36]. Zapico et al [48] argued that reflection on previous behavior helps people prioritize behavior change, and they developed a reflection app [48,52]. Apps can also support prioritization by providing salient reminders of past behavior as memories of past behavior may not be accurate [42]. Reitberger et al [42] supported this by linking a selection app with behavior-monitoring data from a reflection app. This allowed the selection app to assess the suitability of products from the perspective of past behavior.

##### Scope of Impact

Although a benefit of mobile apps lies in their scalability [13], the impact is limited to the users who choose to adopt the technology [9,40]. This is often only a small proportion of the population [40]. However, Reitberger et al [42] noted that this impact can be extended to household members by monitoring household purchases using a reflection app. Reflection on household behavior can spark conversation and lead to social facilitation.

Retailer dependency can also restrict the scope of impact. Information about purchases is available only at specific stores [52]. The app may have little value for people who purchase much of their food from other retailers [49]. The commercial interest of retailers may also lead them to restrict the information that is provided. For example, retailers may be interested in providing data that encourage the purchase of organic products but may be less inclined to provide data on other indicators of sustainability [52]. Retailers may also choose to withhold negative information or withhold information altogether as negative information was found to have a stronger impact on rejecting a product than that of positive information on choosing a product [53].

#### Interaction Challenges

##### Engagement

Engagement issues (limited use and abandonment) were frequently reported [12,13,21,32,45,46,49,51,54-57]. Engagement issues are seen as a barrier to behavioral impact [21,32,46,49,55], and 4% (2/55) of the studies attributed insignificant findings to this issue [46,49]. However, Zapico et al [48] did not observe a correlation between frequency of engagement and behavioral impact. They reported significant behavioral impact despite a low engagement frequency with their App 25 (EcoPanel) reflection app. Engagement issues may result from knowledge gain (learning) [50,58], a perceived burden of app use [46,57,58], performance [13,49], and trust issues [49], whereas tailored or personalized feedback can increase engagement [49,55]—although personalized feedback can negatively affect engagement when the data are not trusted [49]. Normark and Tholander [59] found that, when technology does not perform as expected, this leads to frustration and rejection of the technology.

##### Burden of Use

The use of the technology may be found effortful and disruptive to the shopping routine [42,44,60] or shopping list creation [12] and, therefore, less acceptable for frequent use [58,59]. Instead, studies indicated that technology is used as an infrequent lookup and to explore new products [12,44,58], which may not have been the intended use [12]. However, one study [58] was found to consider the likelihood of infrequent use as part of their design process.

##### Learning

Learning (knowledge gain) was framed as a challenge from the viewpoint of some authors as they perceived that it might hinder repeated use of the app and contribute toward its abandonment. Indeed, as people gain knowledge about products through the use of the app, they may perceive less need to use it again [50,58]. However, knowledge gain (ie, increasing food literacy) through digital interaction was frequently highlighted as an important mediator of behavior change [33,35,36,38,50]. This means that learning was viewed as a challenge for food purchase choice app adoption but not necessarily for achieving behavior change. Moreover, knowledge gain was generalized beyond the products that were evaluated using the app [58] and even the food group [50]. Some authors suggested that, because of rapid learning, initial app interactions may be the most important for guiding change [48,58], and in some cases, further engagements may have little incremental impact on further behavior change [48]. In addition, Bangia et al [38] observed a significant behavioral impact from a one-off intervention with their app 6 months after the intervention had taken place, pointing out that repetition is not an essential mechanism to achieve knowledge gain and mediate behavioral impact.

##### Trust

Issues with trust were also flagged as negatively affecting engagement with technology. Involvement of the retailer in the app source data was noted as a potential source of distrust [12,16,58], leading to efforts to develop technology that can work independently from the retailer [16]. Commercial interests may restrict transparency [52], involve advertisements in the form of recommendations to influence purchases [61], or restrict product information unfavorably [53]. Even when the motives of influence are genuine, external influence may result in reactance (the resistance against a persuasive intent that is nonetheless perceived as manipulation). van der Laan and Orcholska [41] showed how a persuasion attempt to choose a healthier alternative can be nullified when external motives are exposed.

Commercial interests may also make demands on privacy protection, particularly when personal data are shared [44]. As many of the apps are designed to be used in nonprivate environments, privacy concerns are also relevant for the mode of feedback, particularly when the feedback is not well concealed (eg, when using audio feedback [60]) or when the phone camera is used for recognition of products and personal identifiable features may be exposed [36].

#### Technical Feasibility Challenges

##### Source Data

Acquiring and maintaining (accurate) source data may also present feasibility issues, for example, when using location information for recognizing products [44,62,63]. Acquiring and updating source data to achieve the required performance was found to be very challenging [63,64]. In addition, some data, such as data on environmental sustainability and social impact, may be difficult to acquire [36,44,58,65]. This can pose significant implementation challenges.

##### Performance

Technical challenges may stand in the way of the practical implementation of an app. For example, performance issues may limit the app regarding approaches using image recognition. Studies that reported on recognition performance found that not all products could be identified [66,67], and no evaluation studies were found for apps that use image recognition to identify products. Indeed, Isley et al [39] noted that water bottles could not be detected by a camera, and QR labels on shelves were needed to assist their study. They also reported that cereal boxes were not always accurately detected or were not detected at all.

Field trials using near-field communication (NFC) or radiofrequency identification (RFID) as a detection method were not found. Although no performance issues were reported, it was noted that NFC or RFID labels were not (yet) provided for the grocery products in the study supermarket [42], which made practical implementation of this strategy unfeasible at the time of the study.

Even when a product is correctly scanned, product data may not be returned. We found various studies that noted issues with missing or faulty data for products, particularly in the case of retailer-independent designs [46,47,54,55], although faulty data could also be observed for retailer-dependent designs [12].

## Discussion

### Principal Findings

We systematically reviewed the academic literature on food purchase choice apps and inductively charted common design characteristics as well as opportunities and challenges for their effective implementation. We identified 5 distinct app types, each supporting a specific stage of food purchase choice. The most common app type (27/46, 59%) was selection apps. Most selection apps identify products through barcode scanning and evaluate the product by returning a (tailored or norm-based) rating of nutritional content without any dependence on a specific retailer. Other types of apps and product information were less frequent.

We investigated opportunities and challenges by charting quantitative results from evaluation trials and qualitatively exploring patterns in the authors’ observations. Similarly to Chan et al [14], we observed that the results from 11 comparative evaluation trials indicate the potential for food purchase choice apps to support behavior change. However, the proportion of evaluated selection apps is notably small (2/26, 8% vs 9/20, 45% for other types); moreover, the results for this app type are mixed, suggesting potential efficacy challenges with this approach [83,84]. Notably, all comparative evaluations that provided diet balance information (3/11, 27%) showed a positive impact. Evaluation trials also indicated the potential of food purchase choice apps to contribute to knowledge gain, with all 4 evaluations reporting significant findings.

Qualitative findings based on content analysis of the authors’ observations highlighted opportunities and challenges surrounding food purchase choice apps. In food purchase choice apps, engagement issues such as repeated use and long-term adoption present a common challenge. The findings indicate that engagement challenges frequently result from a perceived burden of use. These results are in line with those of the research on the abandonment of behavior change technology [85,86]. We also observed broader challenges relevant to the pursuit of behavior change (eg, people may struggle to identify which efforts to prioritize), trust in retailer motives, and technical feasibility. Learning was observed as a common opportunity derived from the use of food purchase choice apps. Opportunities for addressing various challenges were repeatedly found in apps that apply behavior monitoring (reflection apps).

### Implications

#### Consideration of Mechanism of Technology-Mediated Change in Design Thinking

Selection apps address *the moment* of product selection and appeared particularly popular for supporting food purchase choices. Selection apps provide support just-in-time (at “exactly the right moment”) [55] (see the “just-in-time” intervention literature [87,88]; ie, the moment a choice is translated into action in-store and a product is either added or not added to the shopping basket). There is no need to rely on—potentially inaccurate—memory [42]. Rather than relying on rational thinking, this approach allows the user to be guided by a gentle push (nudge) in the direction of healthy or sustainable choices on the spot [41,55,68] (see the studies by Caraban et al [89], Thaler and Sunstein [90], and Sunstein [91] for frameworks of nudging). As such, the approach aligns with a growing acknowledgment that most decisions during in-store grocery shopping are not made through deliberate, rational decision-making processes but rather through heuristics [69] (“mental shortcuts,” or “snap decisions” based on less conscious evaluations of the available information [90,92]). It can be argued that conscious decision-making is not the design aim of a nudge-driven system, but it can be a side outcome. Along these lines, behavior change beyond a single purchase relies on the repeated exposure to these nudges and on the repeated use of the food purchase choice app that uses nudges to achieve impact.

Despite their claimed benefits, we observed frequent reporting of engagement challenges with selection apps and food purchase choice apps in general. Users perceive the use of selection apps as interruptive or burdensome [46,58,93], leading them to slowly abandon their use over time, which jeopardizes the intended behavior change goal of such apps. These findings align with those of Vhaduri and Prioleau [94], highlighting that infrequent engagement with digital health technology jeopardizes the potential health benefits. Similarly, Ni Mhurchu et al [93] found that the healthiness of food purchases only increased for people who frequently used the selection apps.

Various researchers have attempted to characterize the causes that underlie these engagement issues with digital health applications [85,86,94,95] and proposed design modifications that may improve long-term engagement. Kalnikaité et al [27,92] and Todd et al [26] highlighted the importance of causing minimal disruption to the shopping routine and providing “just enough information, in the right form.” A variety of product identification and evaluation approaches may be driven by the premise of minimal disruption in the selection of apps that we found through our literature search. However, these approaches sustain the focus on nudge-based systems. The questions remain open with regard to improving nudge-based systems enough to accomplish long-term use to achieve behavioral impact.

Related to this, we observed 2 important shortcomings in the attempt to improve behavior change impact by designing food purchase choice apps with the premise of repeated use. First, we found that engagement challenges are common across various domains of digital technology (eg, for just-in-time digital health interventions [96,97], web-based diet interventions [98], self-tracking apps [85,95], wearables [99], and Internet of Things [100]). The widespread presence of engagement issues suggests that they are hard to solve, and the results of efforts to achieve engagement for extended periods are likely to be modest at best. Short periods of engagement may be better understood as the rule rather than the exception [85,95].

Second, the proposal to improve nudge-based systems for improved engagement reflects the prioritization of quantity over quality of engagement. A focus on engagement quantity aligns with a nudge-mediated mechanism of behavior change (ie, the app redirects automatic behavior by changing the choice environment without significant involvement of conscious decision-making [90,92]). However, the findings of our study challenge this prioritization of quantity over quality and suggest that other important mechanisms are at play.

Our findings suggest that learning (knowledge gain) plays an important mediating role in the impact of food purchase choice apps on food purchase choice behavior. In their study with a reflection app, Flaherty et al [69] found that behavioral feedback can spark learning through critical reflection on products and one’s beliefs about products and influence future purchases. Reitberger et al [42] and Zapico et al [48] observed that the use of a reflection app contributed to a positive change in food purchase choice. Also indicative of learning, Bangia et al [38] observed a positive change in food purchase choice 6 months after using an information-rich food purchase choice app during 1 shopping trip.

The mechanism of learning-mediated changes appears to depend more on the quality than on the quantity of food purchase choice app engagements. Few interactions may suffice for learning to happen [38,48,58]. A single interaction may even suffice for a significant impact on behavior [38,48]. In their research with selection apps, Samoggia and Riedel [50] and Herbig et al [58] noted that learning can even reduce the likelihood of future use as the perceived value of the information diminishes, findings that align with those of Epstein et al [95], who found that internalization of the learnings from the use of diet trackers can be a cause of abandonment of the technology as such a mechanism of learning-mediated change can intervene with a mechanism of nudge-mediated change.

Although the described mechanism of learning involves conscious reflection, it does not conflict with an appreciation for the role of heuristics in grocery shopping. Flaherty et al [69] observed that conscious reflection serves to update beliefs that drive heuristics-based food purchase choice. After a period of conscious reflection on purchases, routinized, heuristics-based shopping is continued.

In summary, we observed that there is an important role for learning in behavior change supported by food purchase choice apps and that—contrary to a mechanism that relies on nudging—learning is relatively robust against disengagement as it does not necessarily rely on continued engagement with the app. This has important implications for the design of food purchase choice apps. There are opportunities to design apps that can be effective despite potential abandonment that designers may want to pursue. In the next section, we provide some practical suggestions for designing with learning in mind, along with other suggestions for the future design of food purchase choice apps.

#### Suggestions for Future Design

The purpose of this section is to formulate briefly some practical suggestions for the design of food purchase choice apps that follow from the contents of this review.

##### Optimize for Learning

As discussed in the previous section, learning appears to play a key role in the potential behavioral impact of food purchase choice apps. We propose that the key elements of learning-oriented food purchase choice app design include information clarity, information contextualization (ie, providing informative context to support decisions), and an emphasis on rich information—elements that we observed in the use of functions such as *tips and tricks* and *background information*. Key elements also include design with a view for minimal interactions as opposed to repeated use, design to support reflection on information and rational thinking that extends beyond the app’s use, and design to support user autonomy and gradual abandonment of the app.

This proposal aligns with our observations that behavior monitoring can benefit learning [47,48,69] and that the use of knowledge tips and background information is also associated with knowledge gain [38,58]. Importantly, the optimization of designs for a nudging effect [26,27] may conflict with efforts to optimize designs for learning.

##### Formalize Assumptions of App Use Over Time

Many food purchase choice app designs seem to be developed on the unspoken premise of continued engagement to achieve behavioral impact, although this is not a given. We observed that it is uncommon for designers to articulate expectations regarding the regularity and nature of engagement within the design process (an exception is the study by Herbig et al [58]). However, our findings suggest that early abandonment appears to be common and can have a detrimental impact on the potential of an app to affect behavior. To mitigate unforeseen impact, we suggest that designers view abandonment as a key element of their design strategy and formulate engagement expectations early in the design process. It is recommended that designers include considerations about engagement over time and possible abandonment in design thinking. By bringing transparency into the matter, designers can then develop diverse mechanisms to address abandonment.

##### Overcome Biases and Norms When Selecting Design Characteristics

Although we observed a wide range of design characteristics (app types, functions, and product information), food purchase choice app designers seem biased toward selecting a set of characteristics (see, for instance, selection apps and the use of barcode scanning and normative or tailored ratings of nutritional information). We found that other app types and product information may have more potential. Therefore, we encourage designers to explore other options for the design of selection apps and, broadly, of food purchase choice apps. Some suggestions include the use of diet balance as product information and reflection apps as an app type, which are further addressed in the following sections.

##### Use Diet Balance as Product Information

This product information was commonly associated with significant behavioral impact (see the *Study Outcomes* section). Achieving a balanced diet can be framed as goal setting, which is considered an effective BCT [101-103]. In addition, the simplicity of classifying choices into 5 categories may be beneficial for facilitating behavior change. Goal setting requires a form of monitoring and is, therefore, suitable for reflection apps—for which monitoring is a key function. Goal setting is also compatible with planning apps [35] based on purchase intention monitoring. However, the need to define quantities for the intended purchases may present a barrier [12,36,70], which may make this a less suitable option.

##### Consider Behavior Monitoring (Reflection Apps)

We observed that various opportunities for addressing common challenges with food purchase choice apps are found in reflection apps framed around behavior monitoring. Behavior monitoring is considered one of the more impactful BCTs [101,102]. Behavior monitoring can help prioritize actions [48], which is a common barrier to achieving behavior change. Behavior monitoring can be resistant to engagement issues [48], particularly those related to missing product data [47], as it does not depend on the information of a single product (as opposed to many selection apps and nudging approaches). It also provides opportunities for feedback on diet balance, a type of product information that was commonly associated with significant behavioral impact. Moreover, as reflection is independent of the point of purchase (usually happens after the purchase), the interaction with reflection apps is likely to be less time-pressured than the interaction with those designed to be used during shopping (eg, selection apps and nudging systems), which can provide better learning opportunities.

##### Practical Limitations and Future Opportunities for Reflection Apps

Relevant to the aforementioned, it must be noted that most reflection apps included in this study used loyalty card data or receipt scanning, approaches that facilitated easy data recording [42,47,49,52]. However, practical implementation of these approaches can present difficulties. The applicability of loyalty card data is limited to the people who use loyalty cards, and the availability depends on the retailers that make these data available. Although Swiss law seems to require retailers to make these data available through an application programming interface (API) [49], such regulations may not apply in other countries, which can pose constraints to designers who want to pursue such approaches. Moreover, receipt scanning requires trained machine learning algorithms and may not work optimally [47,52]; however, given that alternatives such as barcode scanning are often perceived as too effortful [85], receipt scanning seems to be a promising avenue for future designs. These limitations can be framed as opportunities for further research for developing effective food purchase choice apps.

### Limitations

We acknowledge several limitations in our decisions for executing and reporting this review. We report these in no specific order.

The use of digital technology for behavior change can raise important ethical concerns [104-106] as these technologies have the potential to be manipulative. This review did not systematically assess the ethical concerns that might be associated with the app designs or the approaches that were taken to mitigate manipulative potential (ie, design approaches that respect individual needs and priorities, such as participatory design [107-109] or user-centered design). Such an assessment was beyond the scope of this study. However, designers of technology must be respectful of individual needs and priorities and follow user-centered approaches.

We did not calculate a meta-statistic estimate of the effects of food purchase choice apps. Appropriate reporting of this statistic relies on the stringent review procedure of a meta-analysis. We provided a simple summary analysis of study findings that provided context for the opportunities and challenges that were reported in the literature. Calculating a meta-statistic would be beyond this means and not our objective, and following the requirements for a meta-analysis would have compromised our ability to report on the objectives that we did formulate. Moreover, with the current body of literature, we see little justification for conducting a meta-analysis to calculate the impact of food purchase choice apps. The variety in reported outcome measures, together with a modest number of studies reporting evaluation results, complicates the calculation of a meta-statistic and restricts the conclusions that can be drawn.

The final charting was performed by only 1 person. This limited the rigor of establishing exact counts of the observed codes. However, the main function of observation counts was to indicate the magnitude of common patterns in designs, opportunities, and challenges. Establishing exact counts for each observation was less important. Importantly, the identification of the common patterns themselves played a superior role in addressing the study objectives. Multiple reviewers were involved in the identification of patterns in the literature.

We acknowledge that the apps could have been described along with additional characteristics. We listed the characteristics that appeared most salient in describing the variations in apps without much interpretation. The apps could have been characterized by mapping techniques from the BCT Taxonomy (BCTT) [110]. However, we had several reasons not to characterize apps using BCTs. First, mapping along the BCTT requires a firm understanding of the various techniques as well as a firm understanding of the intervention that applies the techniques [111]. The comprehensiveness and clarity of app descriptions in the articles varied considerably, and it was not feasible to download and investigate the apps as most were not deployed on app stores. This limited our ability to perform accurate mapping of BCTs, with the potential for misleading interpretations drawn from this mapping. Second, the value of mapping BCTs is expected to be moderate. BCTs have been associated with the efficacy of behavioral intervention [101-103], and counting BCTs in apps is used as a practice to estimate the potential of an app to affect behavior [14,21]. However, several studies failed to find a relationship between the number of applied BCTs and behavioral outcomes [14,112,113]. As a binary mapping approach, mapping along the BCTT does not consider the quality of the BCT implementation or its prominence in the app as a whole. For these reasons, we decided not to prioritize the mapping of BCTs.

### Conclusions

This scoping review examined the design characteristics, opportunities, and challenges of food purchase choice apps. Most food purchase choice apps are designed to help users select healthier products by scanning the barcode and displaying a nutritional rating. The value of this design comes from its potential to influence (nudge) users’ decisions at the point of purchase (just in time), with little demand for conscious decision-making. However, our findings suggest that this design approach does not encourage repeated use and long-term adoption, which limits opportunities for behavior change through nudging. Instead, our results indicate that learning plays an important role in behavior change and that this mechanism is less dependent on continued use. We argue that designs that optimize learning within each interaction will have a better chance of achieving behavior change. This review concludes with design recommendations, suggesting that food purchase choice app designers (1) anticipate the possibility of early abandonment as part of their design process and (2) design apps that optimize the learning experience.

## Appendix

Multimedia Appendix 1 Completed PRISMA-ScR (Preferred Reporting Items for Systematic Reviews and Meta-Analyses extension for Scoping Reviews) checklist.

Multimedia Appendix 2 Overview of the included articles.

Multimedia Appendix 3 Overview of apps.

Multimedia Appendix 4 Function descriptions.

Multimedia Appendix 5 Sankey diagram of app designs grouped by app type, product information, and retailer dependency.

Multimedia Appendix 6 Overview of evaluation studies by product information.

Multimedia Appendix 7 Overview of challenges and opportunities.

## Abbreviations

API: application programming interface
BCT: behavior change technique
BCTT: Behavior Change Technique Taxonomy
NFC: near-field communication
PRISMA-ScR: Preferred Reporting Items for Systematic Reviews and Meta-Analyses extension for Scoping Reviews
RFID: radiofrequency identification

## Data Availability

All data generated or analyzed during this study are included in this published article and its supplementary information files.
