# Supplementary material for: Smartphone Apps for Food Purchase Choices: Scoping Review of Designs, Opportunities, and Challenges
Source: J Med Internet Res. 2024 Mar 6;26:e45904. doi: 10.2196/45904 (PMC10955402; doi:10.2196/45904)
Supplement: Multimedia Appendix 2 [file jmir_v26i1e45904_app2.docx]

| **citation** | **Publisher** | **Year** | **Study type** |
| --- | --- | --- | --- |
| **Abao et al., 2018** | Elsevier | 2018 | design and development + feasibility |
| **Ahn et al., 2015** | ACM | 2015 | design and development + feasibility |
| **Akay et al. 2022** | IEEE | 2022 | design and development |
| **Asikis et al., 2021** | Royal Society Publishing | 2021 | design and development + feasibility + evaluation |
| **Bangia et al., 2017** | Elsevier | 2017 | evaluation |
| **Bird et al., 2013** | ACM | 2013 | design and development + evaluation |
| **Bohné et al., 2015** | ACM | 2015 | design and development |
| **Bomfim & Wallace, 2018** | ACM | 2018 | design and development |
| **Bomfim et al. 2020** | ACM | 2020 | design and development + feasibility + process evaluation |
| **Broll et al., 2013** | IEEE | 2013 | design and development + feasibility |
| **Clear et al., 2012** | ACM | 2012 | design and development |
| **Díaz-Hellín et al., 2015** | Springer | 2015 | design and development |
| **Dorman et al., 2010** | MobiCASE 2009 | 2010 | design and development |
| **Dunford et al., 2014** | JMIR | 2014 | design and development + feasibility |
| **Eyles et al., 2017** | Sage | 2017 | feasibility + evaluation |
| **Eyles et al., 2023** | JMIR | 2023 | feasibility + evaluation |
| **Fagerstrøm et al., 2023** | Routledge | 2023 | Feasibility |
| **Flaherty et al., 2018** | Cambridge University Press | 2018 | app store review |
| **Flaherty et al., 2020** | Elsevier | 2020 | feasibility |
| **Fuchs et al., 2019** | ACM | 2019 | design and development + feasibility |
| **Govoruhina and Nikiforova , 2022** | IEEE | 2022 | design and development |
| **Gutiérrez et al., 2018** | ACM | 2018 | design and development |
| **Harada et al., 2022** | IEEE | 2022 | design and development |
| **Head et al., 2014** | Elsevier | 2014 | design and development |
| **Hedin et al., 2022** | MDPI (sustainability) | 2022 | design and development + feasibility + evaluation + process evaluation |
| **Hegen, 2016** | Association for Information Systems | 2016 | feasibility |
| **Herbig et al., 2018** | ACM | 2018 | design and development + feasibility |
| **Hörmann, L., 2019** | IEEE | 2019 | design and development + feasibility |
| **Isley et al., 2017** | IOP publishing | 2017 | design and development + feasibility |
| **Jayananda, 2018** | IEEE | 2018 | design and development |
| **Kassim, M., 2012** | IEEE | 2012 | design and development |
| **Katzeff et al., 2020** | MDPI | 2020 | design and development + feasibility |
| **Kulpy & Bekaroo, 2017** | IEEE | 2017 | design and development + feasibility |
| **Lawo et al., 2021** | ACM | 2021 | design and development + feasibility |
| **López et al., 2017** | Healthcare informatics research | 2017 | design and development + feasibility |
| **Lurz et al., 2023** | IEEE | 2023 | design and development + feasibility + evaluation |
| **Mahdi et al., 2022** | Elsevier | 2022 | feasibility |
| **Mauch et al., 2018** | JMIR | 2018 | app store review |
| **Mauch et al., 2021** | JMIR | 2021 | feasibility |
| **Mönninghoff et al., 2022** | JMIR | 2022 | design and development + feasibility + evaluation |
| **Normark & Tholander, 2014** | ACM | 2014 | design and development + feasibility |
| **Palacios et al., 2018** | MDPI | 2018 | feasibility + evaluation |
| **Reitberger et al., 2014** | Springer | 2014 | design and development + feasibility + evaluation |
| **Röddiger T. et al., 2018** | ACM | 2018 | design and development |
| **Sackey & Ullmann, 2012** | IEEE | 2012 | design and development |
| **Samoggia & Riedel, 2020** | Elsevier | 2020 | process evaluation |
| **Serhani et al., 2019** | IEEE | 2019 | design and development |
| **Tomlinson, 2008** | IEEE | 2008 | design and development + feasibility |
| **Tsai, et al.  2021** | IEEE | 2021 | design and development |
| **van der Laan & Orcholska, 2022** | Elsevier | 2022 | evaluation |
| **Vintsarevich et al., 2011** | Maxwell | 2011 | design and development + feasibility |
| **Waltner et al., 2015** | Springer | 2015 | design and development + feasibility |
| **Waltner et al., 2017** | Springer | 2017 | feasibility |
| **Wiley et al., 2011** | Springer | 2011 | design and development + feasibility |
| **Zapico et al., 2016** | ACM | 2016 | design and development + evaluation |
