# Supplementary material for: Smartphone Apps for Food Purchase Choices: Scoping Review of Designs, Opportunities, and Challenges
Source: J Med Internet Res. 2024 Mar 6;26:e45904. doi: 10.2196/45904 (PMC10955402; doi:10.2196/45904)
Supplement: Multimedia Appendix 4 [file jmir_v26i1e45904_app4.docx]

| Function | Description |
| --- | --- |
| Profiling | Users can setup a user profile to allow for tailored feedback. This function thus generally coincides with tailored feedback. One of the designs [34] uses behavior tracking (physical activity) to dynamically update the user profile. |
| Shopping list | Choice support is provided around shopping list creation, or the application uses automatic generation of a grocery list based on a personal profile and recommendations for a healthy diet. |
| Live search | A function that allows the user to search amongst products on the shelf by providing information of a multiple products simultaneously. This function can take the form of (a) virtual PI projections on products, seen through the phone camera, or (b) a live-updated list of nearby products. |
| Prompting | The user receives a notification of product information that does not follow a direct request for information. E.g., a pop-up message of healthier alternatives that appears when the user scans the barcode of an unhealthy product when the users scan the barcode as part of a payment process [41]. |
| Educating |  |
| Tips | Some applications provide short messages that contain practical knowledge, with the intention to supporting the user in making deliberate food purchase choices. |
| Background information | Some applications provide the user with background information. For example, if a product rating was provided, the application may provide a breakdown on the data that was used for this rating and how the rating was calculated. Application may also provide links to webpages with further information relevant to the product evaluation. |
| Identifying |  |
| Barcode | Barcode scanning. |
| QR | QR code scanning. |
| Image | Computer vision is used to recognize the product with the phone camera. |
| RFID/NFC | RFID and NFC are electromagnetic communication methods used for contactless data transfer, used e.g., in bank cards. |
| Location based | The location of the smartphone relative to a mapped store layout. |
| Loyalty card | For customers with a loyalty card, purchased items can be identified from digital records of past purchases. |
| Receipt scanning | Using algorithms to recognize the characters and the layout of the receipt. |
| Evaluating |  |
| Descriptive | Statistics or raw numeric data, leaving the value judgement to the user. |
| Normative rating | Evaluation against a standardized norm, like presenting a traffic-light label [40] (a label with four colored circles—each circle is either green (best)/yellow/orange/red (worst)—providing a rating for the fat, saturated fat, sugar, and salt content of the product), or a NutriScore [82] label (a 1-score label that consists of a letter—A to F—and associated color—dark green to dark red—that is based on a standard calculation of nutritional characteristics). |
| Tailored rating | Scores for the product based on a profile entered by the user (see the *profiling* function). The rating can be presented in various forms, examples are a score on a scale from 1 to 10 (e.g., [44]), or an adjusted form of the traffic-light label or NutriScore based of personal data. |
| Comparison | Contrasting the product characteristics to that of one or more other products. |
| Analogues | Using another dimension to help the user put the value in context. For example, expressing calories in jogging minutes [71], or carbon footprint in driving miles [36]. |
| Simulation | An estimation of a future state (e.g., body weight [49]) from consuming the product. |
| Rewards | acknowledgement of goal achievement in form of a virtual trophy. |
| Recommending | A list of alternatives for a selected product, or suggestions in relation to a user profile or behavior data. |
| Monitoring | Tracking purchases and returning statistics, e.g., in the form of progress over time (EcoPanel [9,48,52]), performance against a goal [27,33,35], or by estimating a future body state (FutureMe [49]). Note: this is a distinct function from evaluation. In case of monitoring, there is also a form of evaluation, but this evaluation form is not unique to monitoring. |
