# Supplementary figures and images for: Smartphone Apps for Food Purchase Choices: Scoping Review of Designs, Opportunities, and Challenges
Source: J Med Internet Res. 2024 Mar 6;26:e45904. doi: 10.2196/45904 (PMC10955402; doi:10.2196/45904)

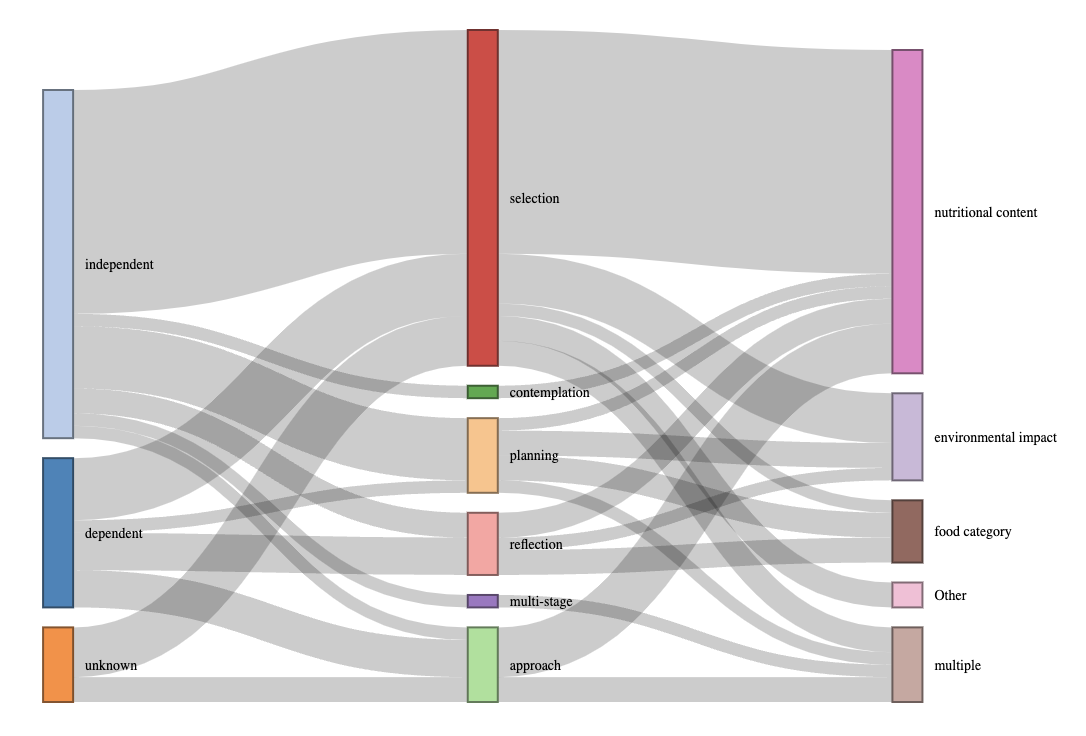

Supplement: Multimedia Appendix 5 [file jmir_v26i1e45904_app5.png]
