# Supplementary material for: Smartphone Apps for Food Purchase Choices: Scoping Review of Designs, Opportunities, and Challenges
Source: J Med Internet Res. 2024 Mar 6;26:e45904. doi: 10.2196/45904 (PMC10955402; doi:10.2196/45904)
Supplement: Multimedia Appendix 6 [file jmir_v26i1e45904_app6.docx]

| **Product Information (PI)** | **App name** | **Citation** | **Design/duration/participants** | **Findings** |
| --- | --- | --- | --- | --- |
| **Nutritional content** | AppX05 | Bangia et al., 2017 | cohort/6 mnt/251p | sig. increase in omega-3-rich purchases. |
|  | AppX13 | Lurz et al., 2023 | cohort/2 wks/31p | n.s. change in the healthiness of purchases. |
|  | Dirk app | van der Laan & Orcholska, 2022 | 3 UI variations (1x with and 2x with health note and 1x without) vs. control/5 wks/1783 scans | (1) sig. healthier purchase when presenting alternatives without health info, (2) n.s. when adding health info. |
|  | Foodswitch | Eyles et al., 2017 | app vs. no app/4 wks/33p + 33p | sig. reduction sodium concentration in purchases. |
|  | Foodswitch | Eyles et al., 2023 | app + reduced sodium salt vs. no app/12 wks/84p + 84p | n.s. change sodium intake. |
|  | FutureMe | Mönninghoff et al., 2022 | app + future-self simulation vs. app without/12 wks/42p + 53p | n.s. difference in the healthiness of purchases. |
| **Food category** | Healthy Shopping App | Bird et al., 2013 | cohort/4 wks/7p | sig. healthier diet balance of purchases. |
|  | MyNutriCart | Palacios et al., 2018 | app vs. education + cohort/8 wks/27p + 24p | sig. healthier diet balance of purchases. |
|  | Nutriflect Home | Reitberger et al., 2014 | cohort/4 wks/21p | sig. healthier diet balance of purchases. |
| **Environmental impact** | EcoPanel | Zapico et al., 2016 | app vs. no app/5 mnt/65p + 2587p | sig. increase organic purchases. |
|  | GreenCobra | Hedin et al., 2022 | cohort/2 wks/30p | n.s. change in the carbon footprint of purchases. |
| **Multiple** | AppX08 | Asikis et al., 2021 | cohort/7 mnt/69p + 323p | sig. increase higher-rated purchases. |
